# Piperacillin/tazobactam for surgical prophylaxis during pancreatoduodenectomy: meta-analysis

**DOI:** 10.1093/bjsopen/zrae066

**Published:** 2024-06-13

**Authors:** Jayant Kumar, Isabella Reccia, Adriano Carneiro, Mauro Podda, Francesco Virdis, Nikolaos Machairas, David Nasralla, Ramesh P Arasaradnam, Kenneth Poon, Christopher J Gannon, John J Fung, Nagy Habib, Omar Llaguna

**Affiliations:** Department of Surgery and Cancer, Hammersmith Hospital, Imperial College London, London, UK; Department of General Surgery, Memorial Healthcare System, Pembroke Pines, Florida, USA; General Surgery and Oncologic Unit, Policlinico ponte San Pietro, Bergamo, Italy; Department of Surgery, Federal University of Pernambuco, Recife, Brazil; Department of Surgery, Calgiari University Hospital, Calgiari, Italy; Dipartimento DEA-EAS, Ospedale Niguarda Ca’ Granda Milano, Milano, Italy; Second Department of Propaedeutic Surgery, National and Kapodistrian University of Athens, Athens, Greece; Department of HPB Surgery, Royal Free Hospital, London, UK; Warwick Medical School, University of Warwick, Coventry, UK; Institute of Precision Diagnostics & Translational Medicine, Coventry, UK; Division of Infectious Disease, Memorial Healthcare System, Pembroke Pines, Florida, USA; Department of General Surgery, Memorial Healthcare System, Pembroke Pines, Florida, USA; Department of Surgery, The Transplantation Institute, University of Chicago, Chicago, Illinois, USA; Department of Surgery and Cancer, Hammersmith Hospital, Imperial College London, London, UK; Department of General Surgery, Memorial Healthcare System, Pembroke Pines, Florida, USA

## Abstract

**Background:**

Pancreatoduodenectomy is associated with an increased incidence of surgical-site infections, often leading to a significant rise in morbidity and mortality. This trend underlines the inadequacy of traditional antibiotic prophylaxis strategies. Hence, the aim of this meta-analysis was to assess the outcomes of antimicrobial prophylaxis, comparing piperacillin/tazobactam with traditional antibiotics.

**Methods:**

Upon registering in PROSPERO, the international prospective register of systematic reviews (CRD42023479100), a systematic search of various databases was conducted over the interval 2000–2023. This inclusive search encompassed a wide range of study types, including prospective and retrospective cohorts and RCTs. The subsequent data analysis was carried out utilizing RevMan 5.4.

**Results:**

A total of eight studies involving 2382 patients who underwent pancreatoduodenectomy and received either piperacillin/tazobactam (1196 patients) or traditional antibiotics (1186 patients) as antibiotic prophylaxis during surgery were included in the meta-analysis. Patients in the piperacillin/tazobactam group had significantly reduced incidences of surgical-site infections (OR 0.43 (95% c.i. 0.30 to 0.62); *P* < 0.00001) and major surgical complications (Clavien–Dindo grade greater than or equal to III) (OR 0.61 (95% c.i. 0.45 to 0.81); *P* = 0.0008). Subgroup analysis of surgical-site infections highlighted significantly reduced incidences of superficial surgical-site infections (OR 0.34 (95% c.i. 0.14 to 0.84); *P* = 0.02) and organ/space surgical-site infections (OR 0.47 (95% c.i. 0.28 to 0.78); *P* = 0.004) in the piperacillin/tazobactam group. Further, the analysis demonstrated significantly lower incidences of clinically relevant postoperative pancreatic fistulas (grades B and C) (OR 0.67 (95% c.i. 0.53 to 0.83); *P* = 0.0003) and mortality (OR 0.51 (95% c.i. 0.28 to 0.91); *P* = 0.02) in the piperacillin/tazobactam group.

**Conclusion:**

Piperacillin/tazobactam as antimicrobial prophylaxis significantly lowers the risk of postoperative surgical-site infections, major surgical complications (complications classified as Clavien–Dindo grade greater than or equal to III), clinically relevant postoperative pancreatic fistulas (grades B and C), and mortality, hence supporting the implementation of piperacillin/tazobactam for surgical prophylaxis in current practice.

## Introduction

Pancreatoduodenectomy (PD) is often fraught with potential complications that can severely impact patient outcomes. Despite significant improvement in the field of surgical care, the postoperative morbidity associated with PD continues to be remarkably high^1^. This highlights a critical area of deficit in patient care that demands further understanding of the challenges that negatively influence postoperative recovery and are congruent with an increase in morbidity and a decline in overall survival. To a large extent, severe perioperative morbidity arises from surgical-site infections (SSIs) and postoperative pancreatic fistulas (POPFs), which affect more than 30% of patients going through this complex surgery^2,3^.

According to the Surgical Care Improvement Project, the necessity to prevent the occurrence of SSIs demands the administration of perioperative antibiotic prophylaxis. However, to achieve optimal efficacy, the choice of antibiotic should effectively target the common bacterial flora present in the biliary tract, comprising enteric Gram-negative bacteria, anaerobes, and enterococci^4^. The Infectious Diseases Society of America, the American Society of Health-System Pharmacists, and the Centers for Disease Control and Prevention support the use of cefazolin, a second-generation cephamycin-type cephalosporin (such as cefoxitin or cefotetan), a third-generation cephalosporin (such as ceftriaxone), or ampicillin/sulbactam as the recommended agent for surgical prophylaxis for procedures involving the biliary tract^5^. Preoperative biliary drainage and the bacterial colonization of the biliary tract underpin the development of bacterobilia and the subsequent development of SSIs and associated major postoperative complications^6,7^.

The causal association between PD and SSIs is not only multifactorial but also challenging to mitigate. For example, the genesis of postoperative intra-abdominal infections is commonly associated with pancreatic anastomotic dehiscence, which may give rise to clinically significant POPFs^8^.

The escalation of antibiotic resistance is due to the proliferation of extended-spectrum β-lactamases, which potentially diminish the efficacy of conventionally prescribed antibiotic agents used in surgical prophylaxis^9^. However, retrospective analyses have highlighted a positive relationship between the administration of a broader-spectrum antibiotic in the perioperative interval and a decline in the rates of infectious complications^5,10^.

The most common microorganisms in bile are *Enterococcus*, *Enterobacter*, *Klebsiella*, and other enteric Gram-negative species. It is imperative to acknowledge that species within the *Enterococcus* and *Enterobacter* genera become resistant to commonly administered prophylactic antibiotics through intrinsic or acquired mechanisms, extending the spectrum of resistance from first-generation to third-generation cephalosporins^9,11^. Therefore, the strategic use of prophylactic antibiotics, tailored according to the resistance pattern of these organisms, may offer a viable approach^12,13^. Further, studies have highlighted that administering broad-spectrum antibiotics, such as piperacillin/tazobactam (PT), substantially reduces overall SSIs.

The primary objective of this meta-analysis was to systematically review the literature and statistically compare the available data to determine the suitability of PT in PD in contrast to the standard antibiotic regimen, intending to reduce SSIs and subsequent complications.

## Methods

### Literature search methodology

This systematic review was performed in accordance with the PRISMA standards^14^. A comprehensive literature search was conducted, incorporating articles catalogued within PubMed, Embase, Web of Science, CINAHL, and clinical trial registries. The search methodology followed was endorsed by the Cochrane Handbook for Systematic Reviews of Interventions and aligned with the reporting criteria for meta-analyses of observational studies in epidemiology^15^. In this study, a comprehensive search strategy was implemented, combining both controlled terms, such as medical subject headings (‘MeSH’) or Embase subject headings (‘Emtree’), and uncontrolled or free terms, namely ‘pancreas’ or ‘pancreatic’, coupled with ‘neoplasm’ or ‘tumor’ or ‘tumors’ or ‘malignancy’, in conjunction with ‘pancreatoduodenectomy’ or ‘pancreatectomy’ or ‘pancreatic surgery’ and ‘antibiotic prophylaxis’ or ‘piperacillin tazobactam’. The intricacies of the search algorithms are outlined in the *Supplementary material*. This study was duly registered in PROSPERO, the international prospective register of systematic reviews (CRD42023479100). A final literature search was performed on 10 November 2023.

The investigation qualified for an exemption from ethical scrutiny because it exclusively employed data from prior publications; likewise, the requirement for informed consent.

### Patient/problem, intervention, comparison, outcome, and study design question

#### Definition

The meta-analysis was structured employing the patient/problem, intervention, comparison, outcome, and study design (PICOS) framework. The focal clinical inquiry assessed was: ‘What is the efficacy of PT as a surgical prophylactic agent in patients undergoing PD when juxtaposed with the standard antibiotic regimen, specifically in terms of reducing SSIs and the attendant complications?’. This query aimed to rigorously evaluate the comparative benefits and potentially mitigative effects of the specified prophylactic antibiotic over the conventional choices, with the ultimate objective of enhancing patient outcomes in the context of complex gastrointestinal surgical procedures.

#### Patient/problem

In this scholarly inquiry, studies were selected that focused on patients undergoing PD, examining the implications of the selected antibiotic prophylaxis on perioperative outcomes. Specifically, the analysis targeted the incidence of SSIs, including superficial, deep, and organ/space infections, as defined by the standardized criteria of the American College of Surgeons National Surgical Quality Improvement Program (ACS-NSQIP) in conjunction with the definitions provided by the Centers for Disease Control and Prevention^16^.

#### Intervention/exposure

This meta-analysis was confined to studies evaluating the administration of PT as surgical prophylaxis in an intervention arm in the context of PD, with a focus on assessing its impact on perioperative outcomes.

#### Comparator/control

The eligibility criteria for comparator studies necessitated the presence of a control cohort administered standard surgical prophylaxis, delineated as a traditional antibiotic (TA) prophylactic regimen. This regimen included administration of one of the following antibiotics: cefoxitin, ceftriaxone, cefazolin, cefmetazole, or ampicillin/sulbactam.

### Inclusion and exclusion criteria for study selection

The previously mentioned searches were completed without restrictions regarding the publication date, type of study, language, or any other delineating parameter. Further, additional studies were confirmed by scrutinizing abstracts, preprints, and the bibliographies of selected papers. Scholarly articles identified as presumably pertinent within the searched databases were organized and transferred to the Reference Manager. Here, redundant entries and duplicates were removed. The titles and abstracts of the remaining articles were independently assessed by two reviewers (J.K. and I.R.). In the case of a dispute, a consensus was reached after arbitration involving one of the chief authors (J.J.F., N.H., or O.L.).

Editorials, case series, narrative reviews, and expert opinions were excluded from the analysis. Articles not written in English or those published without any comparative cohort were also excluded.

### Primary and secondary endpoints

The primary endpoint was the incidence of SSIs, encompassing superficial, deep, and organ/space infections, delineated per the standard interpretation of the ACS-NSQIP and the definitions outlined by the Centers for Disease Control and Prevention^16^.

The secondary endpoints were the incidences of complications, POPFs, delayed gastric emptying (DGE), sepsis, and mortality.

POPFs and DGE were stratified according to the criteria delineated by the International Study Group of Pancreatic Surgery, focusing solely on clinically pertinent instances—specifically, clinically relevant POPFs (grades B and C) and clinically relevant DGE (grades 2–4)^17,18^. The Clavien–Dindo classification system was employed as a standardized framework for reporting and standardizing surgical outcomes within the analysis, with a focus on the identification and assessment of complications classified as Clavien–Dindo grade greater than or equal to III (*Supplementary material*)^19^.

### Data extraction and analysis

From the eligible studies, a range of variables was systematically harvested utilizing a pre-established template by two autonomous reviewers. The included attributes were the first author’s name, the year of publication, the study design and interval, the aggregate sample size, the size of the cohort, any preoperative interventions, including biliary drainage and antibiotics administered, and the incidence of SSIs, morbidity, and mortality^20^. The bias risk assessment for non-randomized study cohorts was carried out utilizing the ROBINS-I tool, whereas the Cochrane risk-of-bias tool was employed for evaluating bias within randomized studies^21,22^.

A meta-analysis of the qualified studies was executed using RevMan software (Review Manager version 5.4; Nordic Cochrane Centre, Copenhagen, Denmark) and the results are displayed as forest plots^23^. Here, the Mantel–Haenszel methodological framework was utilized and both fixed and random-effects models were incorporated to determine the impact of heterogeneity on the analysed outcomes. This approach was incorporated not only to identify the inherent variance but also to assess the impact on obtained results. The degree of heterogeneity among included studies was measured using the *I*^2^ statistic, with values less than or equal to 25% indicating low heterogeneity and those greater than or equal to 75% indicating high heterogeneity^24^.

Data analysis was conducted to detect any anomalous data subset, which, upon identification, was subjected to exclusion from the computation of effect sizes, hence assuring the integrity and robustness of the statistical analysis.

The data sets of quantitative variables were thoroughly analysed to estimate the composite ORs with 95% confidence intervals, comparing PT and traditional/standard antibiotic regimens, whereas the analysis of categorical variables involved the application of the chi-squared test or Fisher’s exact test, which was determined by the data set. The criterion for statistical significance was set at *P* ≤ 0.05. The assessment of prospective publication bias operated under the hypothesis that, in the absence of such bias, larger-scale studies would congregate proximate to the mean effect size, with a symmetrical dispersion of studies around this mean.

## Results

### Characteristics of included studies

**Fig. 1:**
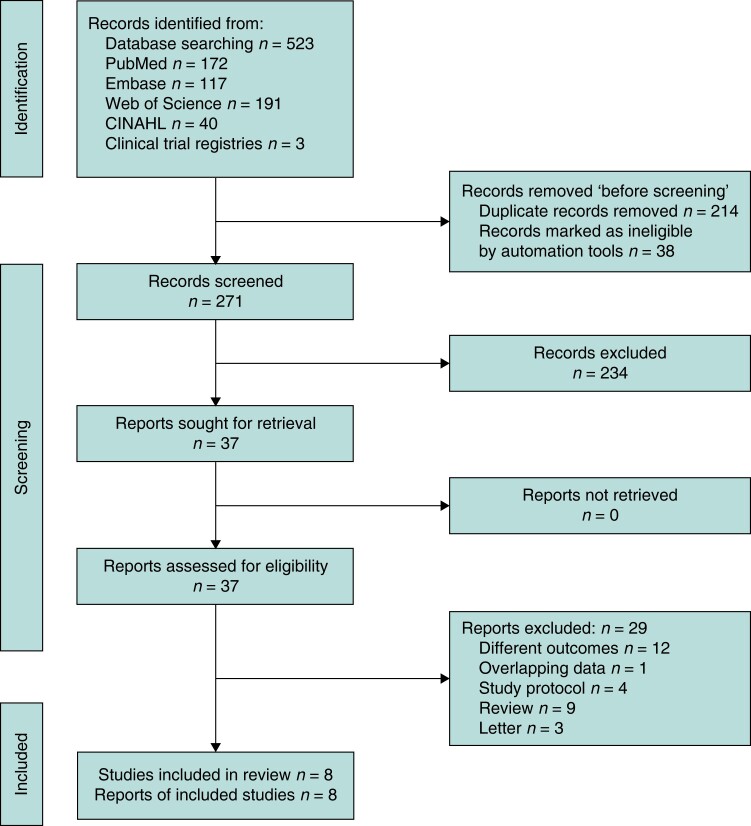
Overview of the search strategy and study selection process following the PRISMA protocol

The preliminary review of the literature yielded 523 studies. After the elimination of duplicates and a thorough review of titles, abstracts, and full texts, a total of eight studies were deemed suitable for inclusion (*Fig. 1*)^4,25–31^.

Out of the eight studies, four were prospective (with two being RCTs) and the remaining four were retrospective. Investigations conducted by Ellis *et al*.^32^ and by D’Angelica *et al*.^25^ pertained to an identical RCT; the latter was incorporated into the analytical framework as it delineated a broader spectrum of endpoints of interest. See *Table 1*.

**Table 1:** Characteristics of included studies

Study	Study design	Study interval	Country	Study arms (*n*)	Age (years), mean(s.d.)	Male
D’Angelica *et al*.^25^ (2023)	RCT	2017–2021	USA and Canada	PT (378)	66.7(10.6)	233 (61.6)
				TA (400) (cefoxitin)	67.2(10.5)	223 (55.7)
Yang *et al*.^26^ (2024)	Retrospective	2018–2022	China	PT (215)	62.4(11.4)	143 (66.5)
				TA (192) (ceftriaxone)	61.1(11.7)	120 (62.5)
Fromentin *et al*.^27^ (2022)	Retrospective	2010–2016	France	PT (81)	66.0(13.6)	50 (61.7)
				TA (65) (cefoxitin)	65.4(13.6)	42 (54.6)
De Pastena *et al*.^28^ (2021)	Retrospective	2015–2018	Italy	PT (296)	64.6(11.2)	163 (55.0)
				TA (383) (ampicillin/sulbactam)	64.6(11.9)	223 (58.2)
Degrandi *et al*.^29^ (2019)	Retrospective	2008–2017	France	PT (69)	67.3(10.6)	42 (60.8)
				TA (53) (cefmetazole)	65.3(10.4)	27 (50.9)
Tanaka *et al*.^30^ (2018)	Prospective	2015–2017	Japan	PT (32)	65.9(26.4)	18 (56.3)
				TA (40) (cefmetazole)	64.3(33.1)	24 (60)
Okamura *et al*.^31^ (2017)	RCT	2008–2017	Japan	PT (19)	66.4(25.6)	NA
				TA (19) (cefazolin)	68.8(24.0)	NA
Donald *et al*.^4^ (2013)	Prospective	2008–2009	USA	PT (106)	63.3(14.4)	49 (46.2)
				TA (34) (cefoxitin/cefazolin/clindamycin)	63.4(14.4)	12 (35.2)

Values are *n* (%) unless otherwise indicated. PT, piperacillin/tazobactam; TA, traditional antibiotics; NA, not available.

The quality assessment tools for cohort and randomized studies showed that the quality of the included studies was low or moderate. See *Tables 2*, *3*.

**Table 2:** Evaluation of risk of bias utilizing the ROBINS-I tool for cohort studies

Study	Confounding	Selection of participants	Classification of intervention	Deviation from intended intervention	Missing data	Measurement of outcomes	Selection of reported results	Overall risk of bias
Yang *et al*.^26^ (2024)	Moderate	Moderate	Moderate	Low	Low	Low	Low	Moderate
Fromentin *et al*.^27^ (2022)	Moderate	Moderate	Moderate	Low	Low	Low	Low	Moderate
De Pastena *et al*.^28^ (2021)	Moderate	Moderate	Low	Low	Low	Low	Low	Moderate
Degrandi *et al*.^29^ (2019)	Low	Moderate	Low	Moderate	Low	Moderate	Low	Moderate
Tanaka *et al*.^30^ (2018)	Low	Moderate	Low	Low	Moderate	Low	Low	Moderate
Donald *et al*.^4^ (2013)	Moderate	Moderate	Low	Low	Moderate	Moderate	Low	Moderate

**Table 3:** Assessment of risk of bias according to the Cochrane tool for randomized studies

Study	Randomization	Deviation from intended intervention	Missing data	Measurement of outcomes	Selection of reported results	Overall risk of bias
D’Angelica *et al*.^25^ (2023)	Low	Low	Low	Low	Low	Low
Okamura *et al*.^31^ (2017)	Low	Low	Moderate	Moderate	Low	Moderate

### Patient population characteristics

A total of eight studies involving 2382 patients satisfied the pre-established selection criteria for inclusion. Of these, 1196 patients were given PT as a prophylactic agent during PD and their outcomes were measured against 1186 patients who had been administered TAs.

Baseline characteristics, including age, sex, BMI, diabetes mellitus, and preoperative biliary drainage, were comparable across the patient groups (*Supplementary material*).

### Primary endpoints

#### Surgical-site infections

**Fig. 2:**
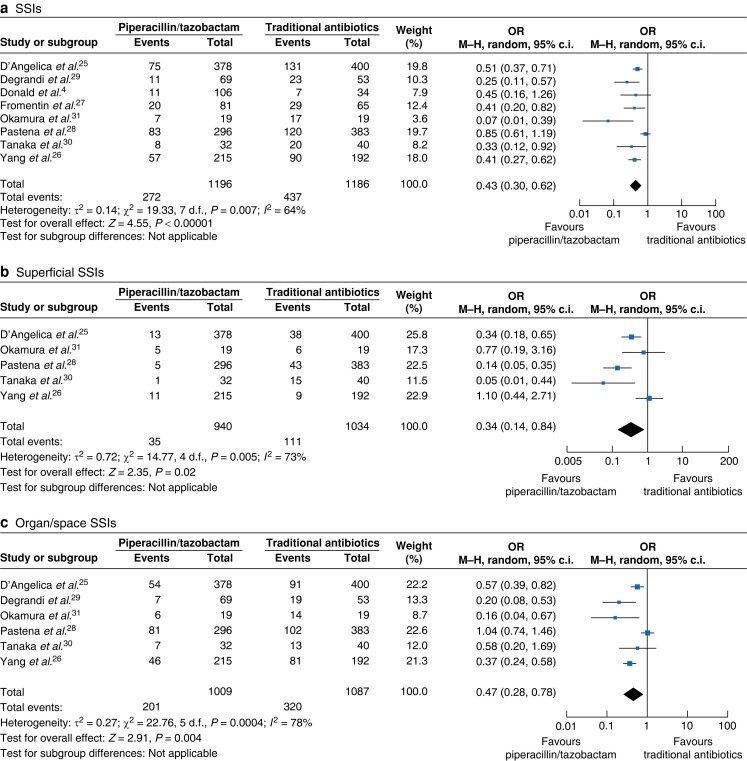
Forest plots demonstrating the incidence of surgical-site infections, superficial surgical-site infections, and organ/space surgical-site infections in patients undergoing pancreatoduodenectomy One group received piperacillin/tazobactam and the other group received traditional antibiotics as surgical prophylaxis. The meta-analysis was conducted utilizing a Mantel–Haenszel random-effects model. The size of the squares depicts the effects, while comparing the weight of the study, a diamond shows favour towards a group, and horizontal bars represent 95% confidence intervals. M-H, Mantel–Haenszel.

The primary outcome measure, illustrated through pooled ORs and their corresponding 95% confidence intervals, was focused on the incidence of SSIs. This comprehensive evaluation determined the incidence of overall, superficial, deep, and organ/space infections, and the results were analysed for patient cohorts who underwent interventions with either PT or TAs, as shown in *Fig. 2*.

A total of eight studies were included in the quantitative analysis involving 2382 patients who underwent PD and demonstrated a significantly lower incidence of SSIs in patients receiving PT as a prophylactic agent (pooled OR 0.43 (95% c.i. 0.30 to 0.62); *P* < 0.00001), with high heterogeneity (*I*^2^ = 62%). The certainty of evidence was considered to be moderate.

Analysis of five studies involving 1974 patients who had superficial SSIs showed a significantly lower incidence of superficial SSIs in the PT group (pooled OR 0.34 (95% c.i. 0.14 to 0.84); *P* = 0.02), with high heterogeneity (*I*^2^ = 73%). The certainty of evidence was considered to be moderate.

Analysis of six studies involving 2096 patients who had organ/space SSIs showed a significantly lower incidence of organ/space SSIs in the PT group (pooled OR 0.47 (95% c.i. 0.28 to 0.78; *P* < 0.004), with high heterogeneity (*I*^2^ = 78%). The certainty of evidence was considered to be moderate.

**Fig. 3:**
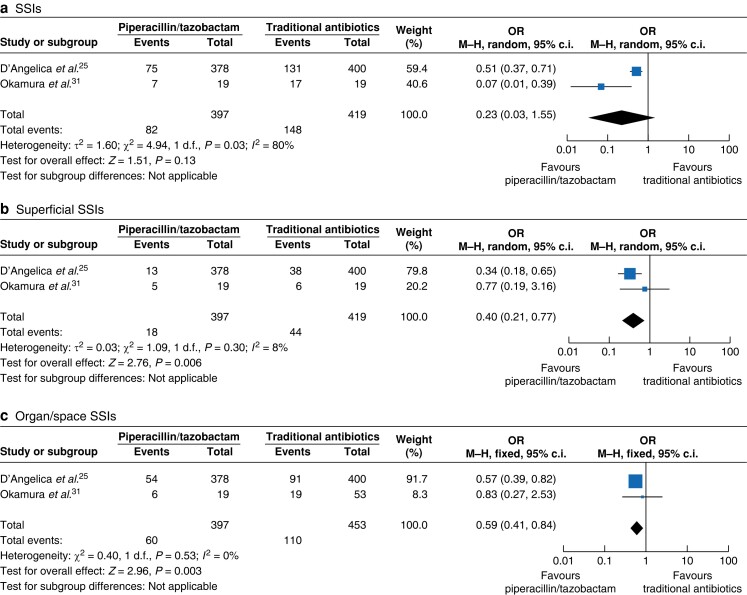
Forest plots of included RCTs demonstrate the incidence of surgical-site infections, superficial surgical-site infections, and organ/space surgical-site infections within a patient cohort undergoing pancreatoduodenectomy One group received piperacillin/tazobactam and the other group received traditional antibiotics as surgical prophylaxis. The meta-analysis was conducted utilizing a Mantel–Haenszel random-effects model. The size of the squares depicts the effects, while comparing the weight of the study, a diamond shows favour towards a group, and horizontal bars represent 95% confidence intervals. M-H, Mantel–Haenszel.

A subgroup analysis of the two RCTs involving 816 patients showed a similar incidence of SSIs in the studied groups (pooled OR 0.23 (95% c.i. 0.03 to 1.53); *P* = 0.13), with moderate heterogeneity (*I*^2^ = 64%). The certainty of evidence was considered to be moderate. However, when the analysis was stratified for superficial and organ/space SSIs, the data indicated lower incidences in the PT group compared with the TA group (pooled OR 0.40 (95% c.i. 0.21 to 0.77); *P* = 0.006 for superficial SSIs and pooled OR 0.59 (95% c.i. 0.41 to 0.84); *P* = 0.003 for organ/space SSIs), with low heterogeneity (*I*^2^ = 8% and 0% respectively). The certainty of the evidence was considered to be low, due to the low number of RCTs available. See *Fig. 3*.

Another subgroup analysis of the six retrospective studies involving 1566 patients showed a significantly lower incidence of SSIs in the studied groups (pooled OR 0.45 (95% c.i. 0.29 to 0.69); *P* = 0.0003), with high heterogeneity (*I*^2^ = 80%). The certainty of evidence was considered to be low, due to the low number of RCTs available. However, when the analysis was stratified for superficial and organ/space SSIs, the data indicated a similar incidence and a lower incidence respectively in the PT group compared with the TA group (pooled OR 0.23 (95% c.i. 0.04 to 1.38); *P* = 0.11 for superficial SSIs and pooled OR 0.48 (95% c.i. 0.24 to 0.97); *P* = 0.04 for organ/space SSIs), with high heterogeneity (*I*^2^ = 85% and 82% respectively). The certainty of the evidence was considered to be low, due to the retrospective design of the studies. See *Table 4*.

**Table 4:** Pooled estimates of primary and secondary endpoints using random-effects meta-analysis for non-randomized studies

Primary or secondary endpoint	Number of studies	PT group	TA group	OR (95% c.i.)	*P*	*I* ^2^ (%)
SSIs	6	190 (23.8)	289 (37.7)	0.45 (0.29,0.69)	0.0003	64
Superficial SSIs	3	17 (3.1)	67 (10.9)	0.23 (0.04,1.38)	0.11	85
Organ/space SSIs	4	137 (10.9)	215 (32.2)	0.48 (0.24,0.97)	0.04	82
Complications classified as Clavien–Dindo grade ≥III	5	89 (12.8)	143 (19.5)	0.61 (0.45,0.81)	0.0008	0
Clinically relevant DGE (grades 24)	3	70 (17.2)	59 (12.5)	1.36 (0.93,1.99)	0.12	0
Clinically relevant POPFs (grades B and C)	5	124 (18.9)	168 (23.9)	0.69 (0.53,0.90)	0.007	0
Sepsis	3	33 (5.6)	85 (13.5)	0.28 (0.09,0.91)	0.03	77
Mortality	3	13 (2.9)	25 (4.9)	0.50 (0.25,1.01)	0.05	0

Values are *n* (%) unless otherwise indicated. PT, piperacillin/tazobactam; TA, traditional antibiotics; SSIs, surgical-site infections; DGE, delayed gastric emptying; POPFs, postoperative pancreatic fistulas.

### Secondary endpoints

#### Complications classified as Clavien–Dindo grade greater than or equal to III

**Fig. 4:**
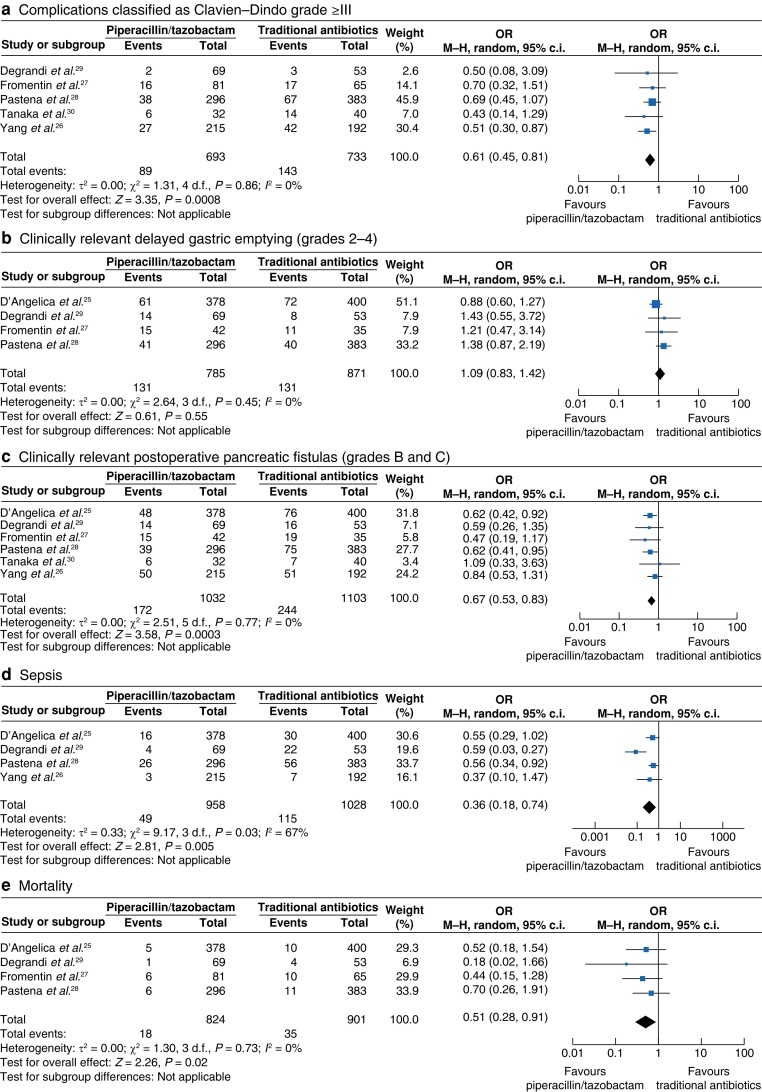
Forest plots demonstrating the incidence of complications classified as Clavien–Dindo grade greater than or equal to III, clinically relevant delayed gastric emptying (grades 2–4), clinically relevant postoperative pancreatic fistulas (grades B and C), sepsis, and mortality in patients undergoing pancreatoduodenectomy One group received piperacillin/tazobactam and the other group received traditional antibiotics as surgical prophylaxis. The meta-analysis was conducted utilizing a Mantel–Haenszel random-effects model. The size of the squares depicts the effects, while comparing the weight of the study, a diamond shows favour towards a group, and horizontal bars represent 95% confidence intervals. M-H, Mantel–Haenszel.

Complications classified as Clavien–Dindo grade greater than or equal to III were documented in five studies involving 1426 patients. The incidence was remarkably less in the PT group (pooled OR 0.61 (95% c.i. 0.45 to 0.81); *P* = 0.0008), with low heterogeneity (*I*^2^ = 0%). The deduced level of confidence in the evidence was moderate. See *Fig. 4*.

A subgroup analysis of included retrospective studies showed a significantly lower incidence of complications classified as Clavien–Dindo grade greater than or equal to III in the studied groups (pooled OR 0.61 (95% c.i. 0.45 to 0.81); *P* = 0.0008), with low heterogeneity (*I*^2^ = 0%). The certainty of evidence was considered to be moderate. See *Table 4* and *Fig. S1*.

#### Clinically relevant delayed gastric emptying (grades 2–4)

Clinically relevant DGE (grades 2–4) was documented in four studies involving 1651 patients. The incidence was found to be similar in both groups (pooled OR 1.09 (95% c.i. 0.83 to 1.42); *P* = 0.55), with low heterogeneity (*I*^2^ = 0%). The deduced level of confidence in the evidence was moderate. See *Fig. 4*.

A subgroup analysis of included retrospective studies showed a similar incidence of clinically relevant DGE (grades 2–4) in the studied groups (pooled OR 1.36 (95% c.i. 0.93 to 1.99); *P* = 0.12), with low heterogeneity (*I*^2^ = 0%). The certainty of evidence was considered to be moderate. See *Table 4* and *Fig. S1*.

#### Clinically relevant postoperative pancreatic fistulas (grades B and C)

Clinically relevant POPFs (grades B and C) were reported in four studies involving 2135 patients. The incidence was significantly less in the PT group (pooled OR 0.67 (95% c.i. 0.53 to 0.83); *P* = 0.0003), with low heterogeneity (*I*^2^ = 0%). The deduced level of confidence in the evidence was moderate. See *Fig. 4*.

A subgroup analysis of included retrospective studies showed a significantly lower incidence of clinically relevant POPFs (grades B and C) in the studied groups (pooled OR 0.69 (95% c.i. 0.53 to 0.90); *P* = 0.007), with low heterogeneity (*I*^2^ = 0%). The certainty of evidence was considered to be moderate. See *Table 4* and *Fig. S1*.

#### Sepsis

Sepsis was reported in four studies involving 1986 patients. The incidence was significantly less in the PT group (pooled OR 0.36 (95% c.i. 0.18 to 0.74); *P* = 0.005), with moderate heterogeneity (*I*^2^ = 67%). The deduced level of confidence in the evidence was moderate. See *Fig. 4*.

A subgroup analysis of included retrospective studies showed a significantly lower incidence of sepsis in the studied groups (pooled OR 0.28 (95% c.i. 0.09 to 0.91); *P* = 0.03), with high heterogeneity (*I*^2^ = 77%). The certainty of evidence was considered to be moderate. See *Table 4* and *Fig. S1*.

#### Mortality

Mortality was reported in four studies involving 1725 patients. The incidence was significantly less in the PT group (pooled OR 0.51 (95% c.i. 0.28 to 0.91); *P* = 0.02), with low heterogeneity (*I*^2^ = 0%). The level of confidence in the evidence was considered moderate. See *Fig. 4*.

A subgroup analysis of included retrospective studies showed a similar incidence of mortality in the studied groups (pooled OR 0.50 (95% c.i. 0.25 to 1.01); *P* = 0.05), with high heterogeneity (*I*^2^ = 0%). The certainty of evidence was considered to be moderate. See *Table 4* and *Fig. S1*.

## Discussion

SSIs have been implicated as the most critical element in association with peril, exerting their influence directly and indirectly through subsequent complications, including complications classified as Clavien–Dindo grade greater than or equal to III, sepsis, clinically relevant DGE (grades 2–4), and clinically relevant POPFs (grades B and C), leading to prolonged hospital stays, readmissions, and increased healthcare expenses.

In contrast to prior reviews on this topic, the index meta-analysis evaluates the feasibility of PT as an agent of surgical prophylaxis during PD in contrast to the currently recommended regimen. The results of this analysis have demonstrated reasonable evidence for the acceptability of PT as a surgical prophylaxis method owing to its ability to produce a significant reduction in the incidence of SSIs.

A growing body of evidence highlights that broad-spectrum antibiotics effectively reduce SSI rates, especially compared with standard prophylaxis agents^33,34^. Similarly, a recent study by Fathi *et al*.^35^ explored the effects of targeted antimicrobials guided by bile cultures and demonstrated a significant decline in therapeutic outcomes, epitomized by an up to 21% reduction in SSIs^33,36,37^.

Moreover, this analysis of secondary endpoints has demonstrated that the PT group showed a clinically significant improvement in clinical parameters (that is decreased incidences of clinically relevant POPFs (grades B and C), complications classified as Clavien–Dindo grade greater than or equal to III, sepsis, and mortality).

The reported incidence of POPFs in recent literature is approximately 15–20% and a substantial number of studies have outlined that POPFs frequently lead to a cascade of additional perioperative complications, which in turn may cause a significant increase in mortality rates, up to 35%^7,34^. The index analysis demonstrated a significant reduction in clinically relevant POPFs (grades B and C). The proliferation of collagenase-producing bacteria, particularly *Enterococcus faecalis*, is frequently implicated in initiating and progressing anastomotic leaks. Hence, the observed reduction in this analysis could be explained owing to a modulatory influence on the anastomotic site, secondary to the introduction of PT in the prophylactic regimen^7,34^. Alternatively, it is possible that giving broad-spectrum antibiotics may improve the clinical severity of biochemical pancreatic leaks. This could mean that serious fistulas become less severe and turn into nearly asymptomatic biochemical leaks^38^.

The reductions in SSIs and postoperative sepsis found in the index analysis may limit the need for further antibiotic treatments. This could translate into improved postoperative courses, leading to shorter hospital stays and fewer readmissions, substantially curtailing healthcare costs and diminishing the likelihood of acquiring *Clostridioides difficile* colitis^39,40^. Hence, strategies to reduce SSIs and postoperative sepsis not only have clinical advantages but also assist significantly in improving the overall efficiency and cost-effectiveness of healthcare systems^41,42^.

DGE is reported by a considerable proportion of patients after PD, ranging from 10% to 45%^43,44^. In the present analysis, no apparent advantage of PT prophylaxis over traditional prophylaxis was identified. The predisposing variables contributing to DGE are varied; they include SSIs, sepsis, POPFs, hormonal mediation secondary to leptin/ghrelin, and surgical reconstruction technique. The exact pathophysiological mechanisms underlying DGE after PD have remained elusive. Hence, future studies are much needed to understand the complex interplay of these variables, addressing them in totality and developing more effective management strategies for DGE in patients undergoing PD^43,45,46^.

There are several limitations regarding the present meta-analysis. First, the included studies encompassed both retrospective and prospective designs, with only two of them being RCTs. This could lead to a potential sources of bias, particularly selection bias, and the influence of differences in clinical practice between the studied cohorts. Second, it is also essential to recognize that the included publications were from state-of-the-art hospitals in high-resource countries. This factor inherently indicates a potential bias towards populations with access to superior and advanced medical care, with less prevalence of infectious disease, which may not be representative of global healthcare scenarios. Third, this review is also limited by the observed heterogeneity among the included studies and in terms of the type of TA prophylaxis utilized; however, these conventional antibiotics belong to the same pharmacological spectrum, limiting the associated bias.

This meta-analysis has demonstrated a significant improvement in the incidence of SSIs, as well as the associated morbidity and mortality. The present evidence from the available literature suggests the inclusion of PT as a prophylactic regimen, providing better perioperative coverage against the organisms that cause SSIs after PD. Consequently, future consensus and guidelines concerning the application of prophylactic antibiotics in the context of PD should consider the inclusion of PT as a viable and advantageous option. However, continued research is needed to determine the optimum protocol for including PT as a surgical prophylactic regimen in the index subset of the population.

## Supplementary Material

zrae066_Supplementary_Data

## Data Availability

All data that support the conclusions of this manuscript are included in the *Supplementary material*.
