# Supplementary material for: Piperacillin/tazobactam for surgical prophylaxis during pancreatoduodenectomy: meta-analysis
Source: BJS Open. 2024 Jun 13;8(3):zrae066. doi: 10.1093/bjsopen/zrae066 (PMC11170489; doi:10.1093/bjsopen/zrae066)
Supplement: zrae066_Supplementary_Data [file zrae066_supplementary_data.zip › Supplementary_Material.docx]

Superbugs vs. Old guard. Piperacillin-tazobactam in surgical prophylaxis during

pancreatoduodenectomy: meta-analysis

Authors: Dr Jayant Kumar, MD, PhD ^1,2^, Dr Isabella Reccia MD^3^, Prof Adriano Carneiro MD PhD^4^, Dr Mauro Podda MD^5^, Dr Francesco Virdis MD^6^, Dr Nikolaos Machairas MD, PhD^7^, Dr David Nasralla MD, PhD^8^, Prof Ramesh P Arasaradnam MD PhD^9^, Dr Kenneth Poon MD^10^, Dr Christopher J Gannon MD^2^, Prof John J Fung MD, Phd^11^, Prof Nagy Habib MD, ChM^1^, Dr Omar Llaguna MD^2^

1. Department of Surgery and Cancer, Hammersmith Hospital, Imperial College London, London W12 0TS, UK.
2. Department of General Surgery, Memorial Healthcare System, Pembroke Pines, FL 33028, USA.
3. General Surgery and Oncologic Unit, Policlinico ponte San Pietro, 24036 Bergamo, Italy.
4. Department of Surgery, Federal University of Pernambuco, [Av Professor Moraes Rego 1235, Recife, PE, 50670-901](https://www.bing.com/ck/a?!&&p=f438ba9565416d22JmltdHM9MTY5OTY2MDgwMCZpZ3VpZD0xYmIxYmMzZC01ZTRmLTZmZjUtMDYzNC1hZmI4NWY1MzZlZGImaW5zaWQ9NTcwNQ&ptn=3&ver=2&hsh=3&fclid=1bb1bc3d-5e4f-6ff5-0634-afb85f536edb&u=a1L21hcHM_Jm1lcGk9MTI3fn5Vbmtub3dufkFkZHJlc3NfTGluayZ0eT0xOCZxPVVuaXZlcnNpZGFkZSUyMEZlZGVyYWwlMjBkZSUyMFBlcm5hbWJ1Y28mc3M9eXBpZC5ZTjc5OTN4NjU5NjczNDMwMzkxNjYxMDkxOSZwcG9pcz0tOC4wNTAzMzU4ODQwOTQyMzhfLTM0Ljk0NTU2ODA4NDcxNjhfVW5pdmVyc2lkYWRlJTIwRmVkZXJhbCUyMGRlJTIwUGVybmFtYnVjb19ZTjc5OTN4NjU5NjczNDMwMzkxNjYxMDkxOX4mY3A9LTguMDUwMzM2fi0zNC45NDU1Njgmdj0yJnNWPTEmRk9STT1NUFNSUEw&ntb=1), Brazil
5. Department of Surgery, Calgiari University Hospital, 09121 Calgiari, Italy.
6. Dipartimento DEA-EAS Ospedale Niguarda Ca’ Granda Milano, 20162 Milano, Italy.
7. 2^nd^ Department of Propaedeutic Surgery, National and Kapodistrian University of Athens, 11527 Athens, Greece.
8. Department of HPB Surgery, Royal Free Hospital, London NW3 2QG, UK
9. Warwick Medical School, University of Warwick, Coventry CV4 7H, UK & Institute of Precision Diagnostics & Translational Medicine, Coventry CV2 2DX, UK
10. Division of Infectious Disease, Memorial Healthcare System, Pembroke Pines, FL 33028, USA.
11. The Transplantation Institute, Department of Surgery, University of Chicago, Chicago, IL 60637, USA.

Corresponding author: Kumar Jayant, MD, PhD

- Department of Surgery and Cancer, Hammersmith Hospital, Imperial College London, London W12 0TS, UK.
- Department of General Surgery, Memorial Healthcare System, Pembroke Pines, FL 33028, USA.

Email- j.kumar@imperial.ac.uk

| **Supplementary Figures and Tables** |  |
| --- | --- |
| Supplementary Table 1. Databases and search strategy. | *page 9* |
| Supplementary Figure 1 | *page 19-22* |

Supplementary Table 1. Databases and search strategy.

|  | Database | Search Term and strategy |
| --- | --- | --- |
| 1 | PubMed | ("Pancreas"[MeSH Terms] OR "pancreatic") AND ("Neoplasms"[MeSH Terms] OR cancer OR cancers OR neoplasm OR malignant OR malignancies OR carcinoma OR carcinomas OR tumor OR tumors)  AND  ("Antimicrobial Prophylaxis"[MeSH Terms] OR "antimicrobial prophylaxis" OR "prophylaxis, antimicrobial" OR “antibiotic prophylaxis” OR "Piperacillin-Tazobactam"[MeSH Terms] OR "piperacillin tazobactam" OR piperacillin AND tazobactam)  AND  ("Pancreatectomy"[MeSH Terms] OR "pancreatectomy" OR "pancreatoduodenectomy" OR "duodenopancreatectomy" OR "pancreatic surgery")  AND  ("Bacterobilia"[MeSH Terms] OR "biliary drainage" OR bacteri* OR microbiology* OR "Enterococcus"[MeSH Terms] OR "Enterobacter"[MeSH Terms] OR "enterococcus" AND "enterobacter" OR “Extended-Spectrum Beta-Lactamases” OR “ESBLs” OR antibiotic* OR antimicro*) |
| 2 | Embase | TS=("pancreas" OR "pancreatic")  AND  TS=("neoplasm" OR "cancer" OR "cancers" OR "malignant" OR "malignancies" OR "carcinoma" OR "carcinomas" OR "tumor" OR "tumors")  AND  TS=("antimicrobial prophylaxis" OR "prophylaxis, antimicrobial" OR “antibiotic prophylaxis” OR "piperacillin tazobactam" OR piperacillin AND tazobactam)  AND  TS=("pancreatectomy" OR "pancreatoduodenectomy" OR "duodenopancreatectomy" OR "pancreatic surgery")  AND  TS=("bacterobilia" OR "biliary drainage" OR "bacteri*" OR "microbiology*" OR "Enterococcus" OR "Enterobacter" OR “enterococcus AND Enterobacter” OR “Extended-Spectrum Beta-Lactamases” OR “ESBLs” OR "antibiotic*" OR "antimicro*") |
| 3 | Web of Science | TS=("pancreas" OR "pancreatic")  AND  TS=("neoplasm" OR "cancer" OR "cancers" OR "malignant" OR "malignancies" OR "carcinoma" OR "carcinomas" OR "tumor" OR "tumors")  AND  TS=("antimicrobial prophylaxis" OR "prophylaxis, antimicrobial" OR “antibiotic prophylaxis” OR "piperacillin tazobactam" OR piperacillin AND tazobactam)  AND  TS=("pancreatectomy" OR "pancreatoduodenectomy" OR "duodenopancreatectomy" OR "pancreatic surgery")  AND  TS=("bacterobilia" OR "biliary drainage" OR "bacteri*" OR "microbiology*" OR "Enterococcus" OR "Enterobacter" OR enterococcus AND enterobacter OR “Extended-Spectrum Beta-Lactamases” OR “ESBLs” OR "antibiotic*" OR "antimicro*") |
| 4 | Scopus | (TITLE-ABS-KEY ("pancreas" OR "pancreatic"))  AND  (TITLE-ABS-KEY ("neoplasm" OR "cancer" OR "cancers" OR "malignant" OR "malignancies" OR "carcinoma" OR "carcinomas" OR "tumor" OR "tumors"))  AND  (TITLE-ABS-KEY ("antimicrobial prophylaxis" OR "prophylaxis, antimicrobial" OR “antibiotic prophylaxis” OR "piperacillin tazobactam" OR piperacillin AND tazobactam))  AND  (TITLE-ABS-KEY ("pancreatectomy" OR "pancreatoduodenectomy" OR "duodenopancreatectomy" OR "pancreatic surgery"))  AND  (TITLE-ABS-KEY ("bacterobilia" OR "biliary drainage" OR "bacteri*" OR "microbiology*" OR "Enterococcus" OR "Enterobacter" OR enterococcus AND enterobacter OR “Extended-Spectrum Beta-Lactamases” OR “ESBLs” OR "antibiotic*" OR "antimicro*")) |
| 5 | CINAHL | MH "Pancreas" OR "pancreatic"  AND  MH "Neoplasms+" OR AB, TI "cancer" OR AB, TI "cancers" OR AB, TI "malignant" OR AB, TI "malignancies" OR AB, TI "carcinoma" OR AB, TI "carcinomas" OR AB, TI "tumor" OR AB, TI "tumors"  AND  MH "Antimicrobial Prophylaxis+" OR AB, TI "antimicrobial prophylaxis" OR AB, TI "prophylaxis, antimicrobial" OR AB, TI “antibiotic prophylaxis” OR MH "Piperacillin-Tazobactam Combination+" OR AB, TI "piperacillin tazobactam" OR AB, TI "piperacillin" AND AB, TI "tazobactam"  AND  MH "Pancreatectomy+" OR AB, TI "pancreatectomy" OR AB, TI "pancreatoduodenectomy" OR AB, TI "duodenopancreatectomy" OR AB, TI "pancreatic surgery"  AND  MH "Bacterobilia+" OR AB, TI "biliary drainage" OR AB, TI "bacteri*" OR AB, TI "microbiology*" OR MH "Enterococcus+" OR MH "Enterobacteriaceae+" OR AB, TI "enterococcus" OR AB, TI "enterobacter" OR MH “Extended-Spectrum Beta-Lactamases” OR , AB, TI “ESBLs”, OR AB, TI "antibiotic*" OR AB, TI "antimicro*" |

A. Clavien-Dindo≥3


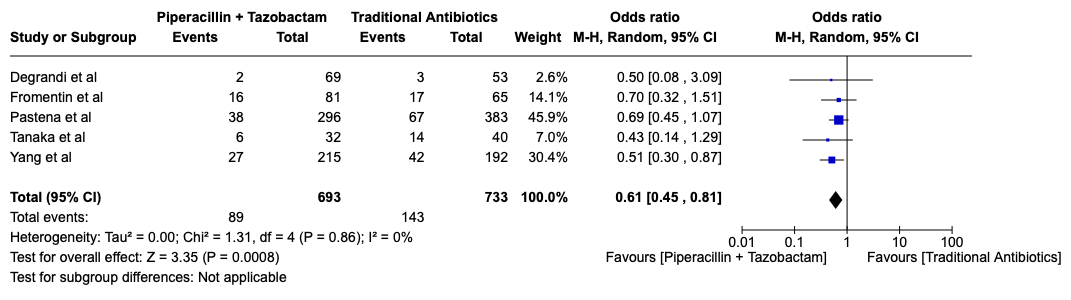


B. Clinically relevant DGE


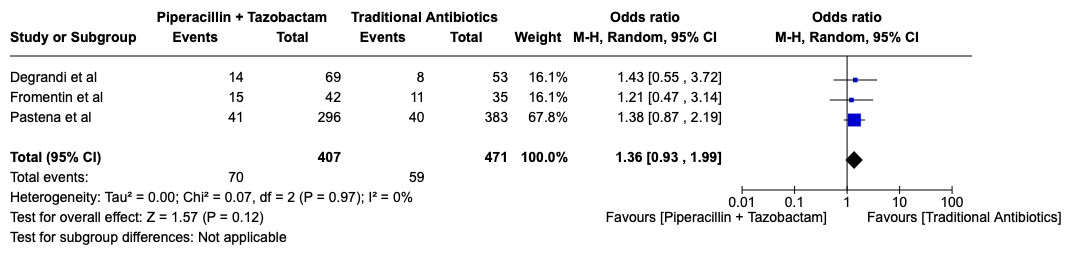


C. Clinically relevant POPF


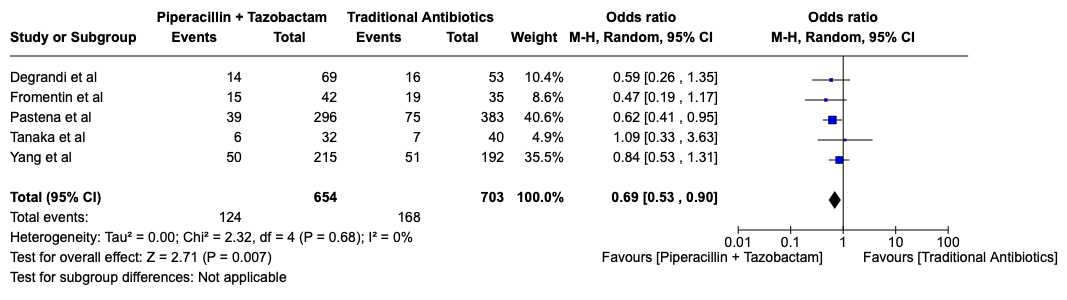


D. Sepsis


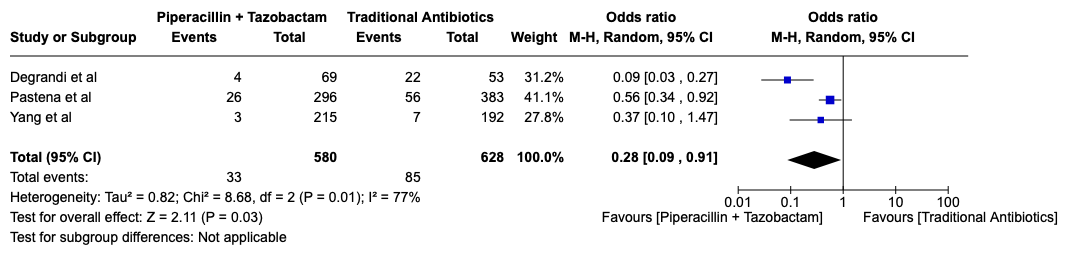


E. Mortality


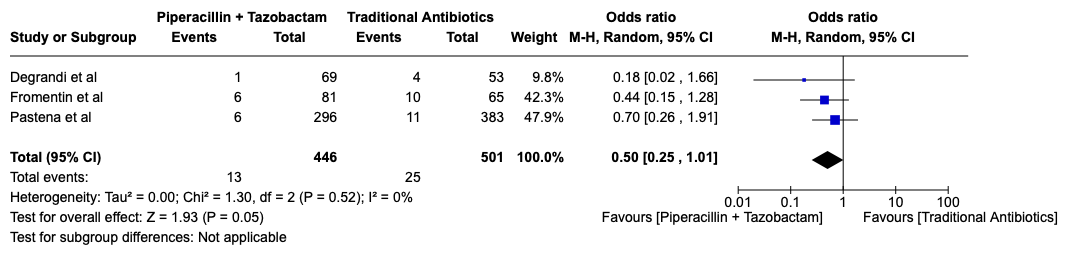


Supplementary Figure 1. Forest plots demonstrate the incidence of Clavien-Dindo ≥3, clinically relevant delayed gastric emptying (DGE, grade 2-4), clinically relevant post-operative pancreatic fistula (POPF, grade B&C), sepsis and mortality within a patient cohort with pancreaticoduodenectomy for included retrospective studies only. Here, one group received “piperacillin-tazobactam” while the other was administered "Traditional Antibiotics" as surgical prophylaxis. The meta-analysis was conducted utilizing a Mantel–Haenszel random effect model. The size of the squares depicts the effects while comparing the weight of the study. The diamond shows a favour towards the group. A 95% confidence interval is represented by horizontal bars.
